# Time-dependent modulation of FoxO activity by HDAC inhibitor in oncogene-transformed E1A+Ras cells

**DOI:** 10.3934/genet.2018.1.41

**Published:** 2018-02-08

**Authors:** Alisa Morshneva, Olga Gnedina, Svetlana Svetlikova, Valery Pospelov, Maria Igotti

**Affiliations:** Institute of Cytology, Russian Academy of Sciences, St. Petersburg, Russia

**Keywords:** histone deacetylase inhibitor (HDACi), sodium butyrate, FoxO, oxidative stress, ROS, Akt, nuclear shuttling

## Abstract

HDAC inhibitors (HDACIs) induce irreversible cell cycle arrest and senescence in mouse embryonic fibroblasts transformed with E1A and c-Ha-Ras oncogenes (E1A+Ras cell line). The aging rate has been associated with the production of high levels of Reactive Oxygen Species (ROS). Specific increases of ROS level have been demonstrated as potentially critical for induction and maintenance of cell senescence process. It's known that HDACs regulate the ROS-dependent FoxO factors, which are responsible for cell growth, proliferation, and longevity. The characteristic ROS increase during aging may be responsible for the decreased HDAC activity, which facilitates the senescent-like phenotype. The objective of this study was to investigate the impact of FoxO transcription factors on HDACIs-induced senescence of E1A+Ras oncogenes transformed cells. This study shows the specific time-dependent effect of HDACI sodium butyrate treatment on FoxO proteins in E1A+Ras cells. Indeed, short-term treatment with NaB results in FoxO activation, which takes place through nuclear translocation, and accompanied by accumulation of such ROS scavengers as MnSOD and SOD2. However, prolonged treatment leads to extensive FoxO degradation and increased intracellular levels of ROS. This degradation is connected with NaB-induced activation of Akt kinase. All of these findings establish that one of the possible mechanism involved in NaB-induced senescence of transformed cells is mediated through down-regulation of FoxO transcription factors and ROS accumulation.

## 1. Introduction

Diversity of FoxO functions is impressively wide and comprises such aspects of cell life as metabolism, DNA repair, cell death, senescence etc. This diversity arises from multi-level regulation, which in turn is greatly dependent on the cell context. That makes regulatory mechanisms of FoxO proteins intricate and to some extent contradictory.

Acetylation, like the rest of post-transcriptional modifications, is an essential part of FoxO regulation. FoxO proteins bind to coactivators and corepressors causing changes in FoxO acetylation level. It is worth noting that many acetylation sites are positioned within DNA-binding region, therefore acetylation in most cases suppress DNA-binding [1]. However, acetylation and deacetylation of FoxO have complex effect on the transcription of target genes and this effect seems to be promoter-specific. It has been revealed that enzymes participating in acetylation of FoxO are also involved in acetylation of histones [2]. That explains the influence of histone deacetylases on FoxO signaling. HDACs were shown to increase FoxO activity inducing its nuclear translocation, while HDAC inhibitors prevent raising the nuclear FoxO level [3]. On the other hand, it should be also mentioned that deacetylation can facilitate FoxO degradation, since the same lysine residues are used for acetylation and polyubiquitination [4].

HDACs was furthermore reported to recover FoxO activity under the stress conditions, since oxidative stress ultimately results in FoxO inhibition by acetylation. Oxidative stress initially leads to FoxO phosphorylation and subsequent translocation to the nucleus, but then activated acetylases repress nuclear activity of FoxO [5]. These post-transcriptional modifications appear to be induced by changes in ROS level [2]. FoxO target genes include such antioxidant genes like catalase, MnSOD, SOD-2, etc [6]. Meanwhile, transcription of target genes depends on the severity of stress. Low levels promote transcription of DNA-repairing and ROS-protective genes, whereas high levels trigger activation of pro-apoptotic genes [7].

The study has been performed on cells transformed by E1A and Ras, both of which are involved in FoxO signaling pathways. E1A stabilizes FoxO by preventing its ubiquitin-dependent proteolysis [8]. At the same time, E1A, being CBP inhibitor, causes repression of FoxO target genes. One possible explanation for this duality is the dual role of CBP, working both as a potent FoxO cofactor and as an acetylase inhibiting FoxO [9]. Ras, the second delivered gene, is involved in many signaling pathways, including PI3K-PKB/Akt and MAPK cascades [4]. As part of PI3K-PKB/Akt pathway, Akt is constitutively active in Ras-transformed cells and stimulates cell growth, inhibition of apoptosis and intense proliferation without signs of density-dependent inhibition, making transformed cells similar to cancerous cells. Ras was also shown to regulate phosphorylation of Forkhead proteins through Ras-Ral signaling [10]. Interestingly, normal activation of Ral enhances FoxO activity, whereas Ras-induced activation of Ral results in FoxO inhibition through activating Akt. In addition, Ras activates three MAPKs: Erk, p38 and JNK [5]. The first two kinases mentioned above have inhibitory effect on FoxO, while JNK activates FoxO in stress condition, triggering nuclear translocation [6].

It should be also mentioned that NaB affects E1A+Ras-transformed cells in a special way. Sodium butyrate, like another HDACIs, is commonly known as apoptosis-inducing agent. This effect of NaB has been demonstrated on a wide range of cell lines, for example nasopharyngeal carcinoma cells [11], hepatic cancer cells [12], ovarian cancer cells [13], etc. E1A+Ras-transformed cells are incapable of undergoing apoptosis under NaB treatment. This fact can be explained with constitutive activity of Ras/PI3K/Akt pathway, which in turn activates the potent antiapoptotic factor NF-κB [14]. Thus NaB in E1A+Ras-transformed cells causes irreversible cell cycle arrest at G1-S phase and senescence, but doesn't lead to apoptosis [15],[16].

The objective of this study was to investigate the impact of FoxO transcription factors on HDACIs-induced senescence of E1A+Ras oncogenes transformed cells.

The paper reveals the FoxO transcription factors role in antiproliferative effects of HDACI sodium butyrate in E1A+Ras-transformed cells. We established time-dependent regulation of FoxO expression by NaB, which was accompanied time-dependent ROS accumulation. The overall aim of the study is to reveal specificity of treatment response in E1A+Ras-transformed cells, constituting a model of cancer cells with overactivation of Ras/PI3K/Akt, which are one of the most difficult to treat. Knowing molecular features of this response can lead us to better treatment solutions.

## 2. Materials and methods

### 2.1. Cell lines

The study has been produced with the use of the stably transformed cells, derived from mouse embryonic fibroblasts by the transfer of complementing oncogenes *E1A* and *cHa-Ras* (line E1A+Ras). HEK-293 cells were kindly provided by Dr. van der Eb. Cells were cultured in high-glucose Dulbecco's modified Eagle's medium (DMEM) with the addition of 10% fetal bovine serum and antibiotics. Cells were treated with 4 mM sodium butyrate (NaB) or 2.5 µM suberoylanilide hydroxamic acid (SAHA) for 24–72 hours (Sigma-Aldrich).

### 2.2. Immunoblotting

Cells were washed in PBS and then lysed. The protein concentration of each cell lysate was determined and equated. Prepared samples were boiled in sample buffer at 100 °C for 5 minutes. Samples were loaded in SDS-PAGE gel and the gel ran at 140–160 V. After electrophoresis proteins were transferred to the PVDF membrane. Membranes were blocked for 1.5 h at room temperature using blocking buffer (5% nonfat dry milk) and incubated with primary antibodies for 1 h at room temperature or overnight at +4 °C. Then membranes were incubated with HRP-conjugated secondary antibodies diluted in blocking buffer for 1h at room temperature. As primary antibodies, we used antibodies to Foxo1 #2880, Foxo3a #2497, phospho-Foxo3a #9466, Akt #9272, phospho-Akt #4060 (Cell Signalling).

### 2.3. RNA isolation and RT-PCR

RNA was isolated from E1A+Ras cells using a TRIzol-based method. Amount of RNA used for generating of cDNA template was 3 mkg. RT-PCR has been performed using primers (100 ng) to the following genes: SOD2 5′-GGCCAAGGGAGATGTTACAAC-3′/5′-CTGAAGGTAGTAAGCGTGCTCC-3′; MnSOD 5′-GCACTGAAGTTCAATGGTGG-3′/5′-ACTGAAGGTAGTAAGCGTGCTC-3′. Quantification of gene expression has been made using the relative standard-curve method and all data was normalized to the absolute control group and subsequently normalized to the gene expression.

### 2.4. Immunofluorescence

Cells were grown on cover glasses, and then fixed in 3.7% formalin for 15 minutes at room temperature, washed in PBS for 10 minutes and in 0.15 M glycine for 15 minutes. After fixation cells were permeabilized in 0.2% Triton X-100 solution and blocked in 3% BSA/PBS for 1.5 hours. Cells were incubated in diluted in the blocking buffer primary antibodies to FoxO1 (#2880P) and FoxO3 (#2497S) overnight at 4 °C and then stained with the anti-rabbit secondary antibodies, conjugated with Alexa Fluor (488) for 1 hour at room temperature. The coverslips were mounted with ProLong Gold Antifade with DAPI.

Images were analyzed and images captured using the Confocal Laser Scanning Microscope Olympus FV3000 (60x, zoom 2.5).

### 2.5. ROS detection

The cell membrane permeant agent 2′,7′-dichlorofluorescin diacetate (DCFDA) was used for detection of reactive oxygen species (ROS). After diffusion into a cell DCFDA undergoes acetylation forming non-fluorescent chemical compound, which can be subsequently oxidized to 2′,7′-dichlorofluorescin (DCF). DCF is a fluorescent compound that can be detected using the method of fluorescent spectroscopy. Cells were washed in warm PBS and incubated for 20 minutes at 37 °С in 5 mkM DCFDA/PBS. After incubation cells were washed in warm PBS and analyzed on flow cytometer Coulter Epicks XL (Bechman, USA) using an excitation wavelength of 488 nm.

## 3. Results

### 3.1. Prolonged NaB administration inhibits FoxO proteins expression and increases the intracellular ROS level

**Figure 1.**
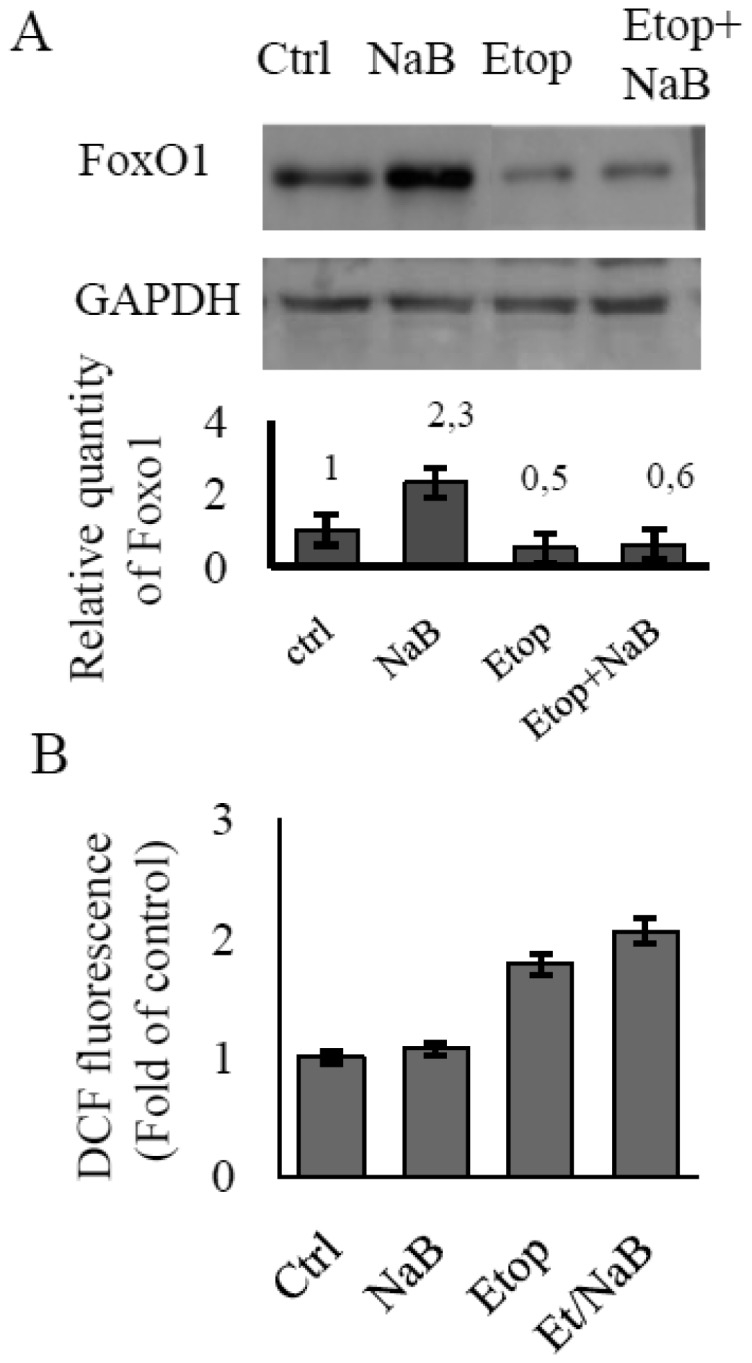
The dynamics of ROS generation in response to etoposide and NaB treatment in E1A+Ras transformed cells. (A) Immunoblotting staining showing FoxO1 expression level in cells being treated with different agents separately or together (2.5 µM etoposide, 4 mM NaB) for 24 h. Expression of Gapdh was used as loading control. The histogram under the immunoblot figure represents the calculation of WB bands intensity in relation to intensity in control point (Ctrl). (B) ROS levels as measured by FACS analysis after staining with DCFDA. The augmentation of median X meaning due to increased fluorescence indicates an increase in the intracellular levels of ROS. The results are presented as the ratio of mean DCF fluorescence at each experimental point to the mean fluorescence in control untreated cells. DCF fluorescence in untreated cells is taken as unit. Histogram displays changes in ROS level in cells being treated as mentioned above (A). Results represent the mean values of three independent experiments, and error bars show the standard deviation. P < 0.05.

The accumulation of ROS has been shown to be in inverse proportion to the level of FoxO1 protein. Etoposide is a well-known cytotoxic agent that causes DNA-damages through an inhibition of topoisomerase II activity [17]. DNA-damaging agents reinforce the production of ROS, so we used etoposide treatment to examine the FoxO level at increased ROS formation. E1A+Ras cells were treated with etoposide alone or in combination with NaB. Immunoblotting data reveal that the least intense FoxO signal belongs to cells being treated with etoposide alone or NaB/etoposide combination for 24 h (Figure 1A). As a consequence, FoxO-dependent containment of the ROS level decreases in response to etoposide as well as to NaB/etoposide. We measured cellular ROS levels in E1A+Ras cells treated without or with etoposide or NaB alone, or in combination etoposide/NaB by FACS-based quantification of fluorescent 2,7-dichlorofluorescein diacetate. As shown in Figure 1B, cellular ROS levels were barely elevated in E1A+Ras cells being treated with NaB for 24 h, because of the potent FoxO activation. In contrast, marked accumulation of ROS was observed in E1A+Ras cells treated with etoposide as well as combination etoposide/NaB, accompanied by an inhibition of FoxO expression (Figure 1B).

**Figure 2.**
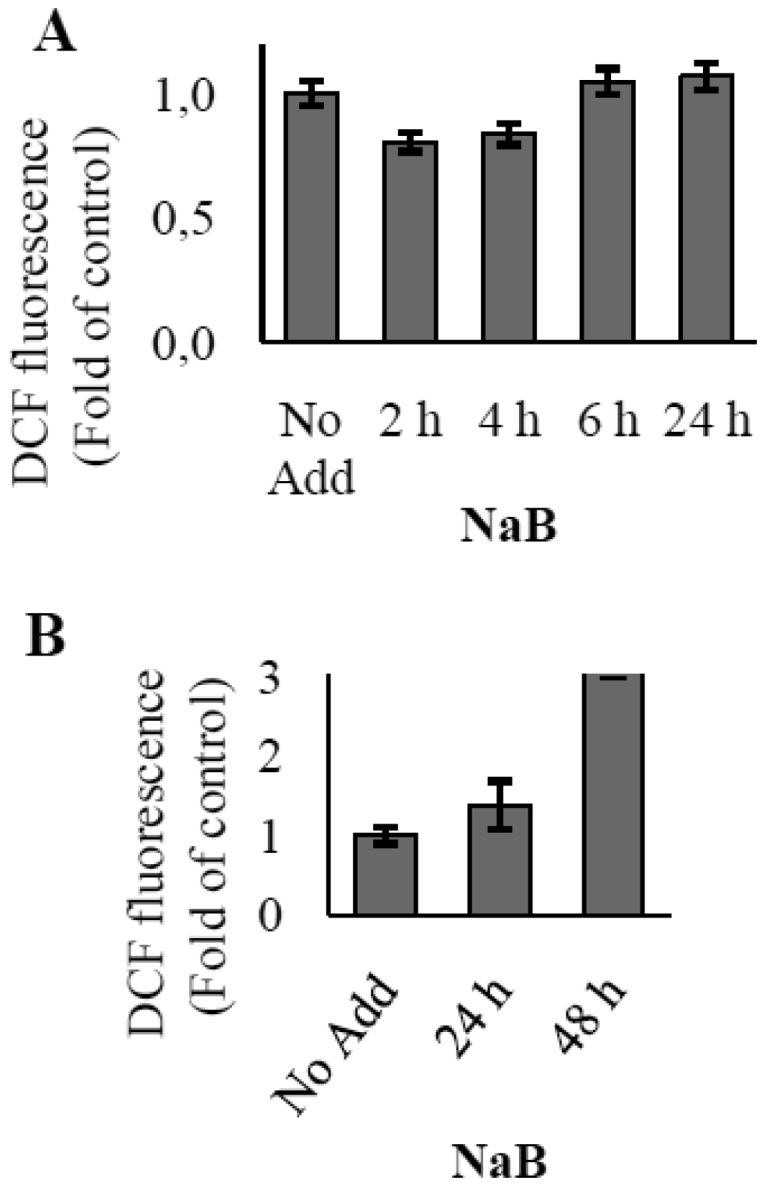
The time-dependent ROS generation in E1A+Ras transformed cells treated with NaB. (A,B) Histograms display dynamics of ROS accumulation during time-course treatment with 4 mM NaB. ROS levels were measured by FACS analysis after staining with DCFDA. The augmentation of median X meaning due to increased fluorescence indicates an increase in the intracellular levels of ROS. The results are presented as the ratio of mean DCF fluorescence at each experimental point to the mean fluorescence in control untreated cells. DCF fluorescence in untreated cells is taken as unit. Results represent the mean values of three independent experiments, and error bars show the standard deviation. P < 0.05.

**Figure 3.**
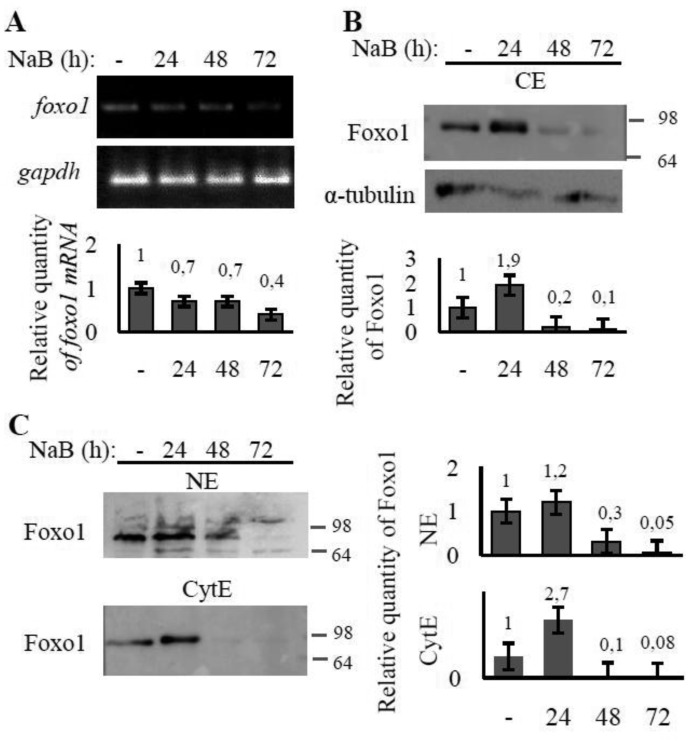
The effect of NaB treatment on FoxO1 expression and localization in E1A+Ras cells. The cells were treated with 4 mM NaB for 24–72 h whilst one well was left untreated (-) and used as a control. (A) RT-PCR analysis of *foxO1*. Transcription of *gapdh* gene was used as loading control. The histogram under the electrophoresis figure represents the calculation of PCR bands intensity in relation to intensity in control point (-). (B) Immunoblotting of proteins performed on total (CE) extracts of E1A+Ras cells using antibodies against Foxo1. (C) Immunoblotting of proteins performed on nuclear (NE) and cytoplasmic (CyE) extracts with antibodies against Foxo1. Expression of α- tubulin was used as loading control. The histograms represent the calculation of WB bands intensity in relation to intensity in control point (-).

It should be mentioned, that NaB alone is also capable to affect the ROS level in time-dependent manner. Short-term treatment with NaB didn't lead to any significant increase in the ROS level (Figure 2A). However, general level of ROS has been shown to increase during prolonged NaB treatment (Figure 2B) with the sharp rise after 48 hours of treatment. Furthermore, immunoblotting data show that accumulation of FoxO1 takes place at a short-term action of NaB no longer of 24 hours. While a longer treatment up to 48 hours results in inhibition of FoxO1 expression (Figure 3B).

Other class of HDAC inhibitor such as SAHA have been used to demonstrate the specificity of NaB-dependent inhibition of FoxO expression. We have observed the same effect of SAHA on FoxO1 expression (Figure S1). Our results indicate as well that SAHA stimulates cellular ROS accumulation in E1A+Ras transformants in time.

### 3.2. Regulation of the FoxO protein level by NaB is primarily post-transcriptional

**Figure 4.**
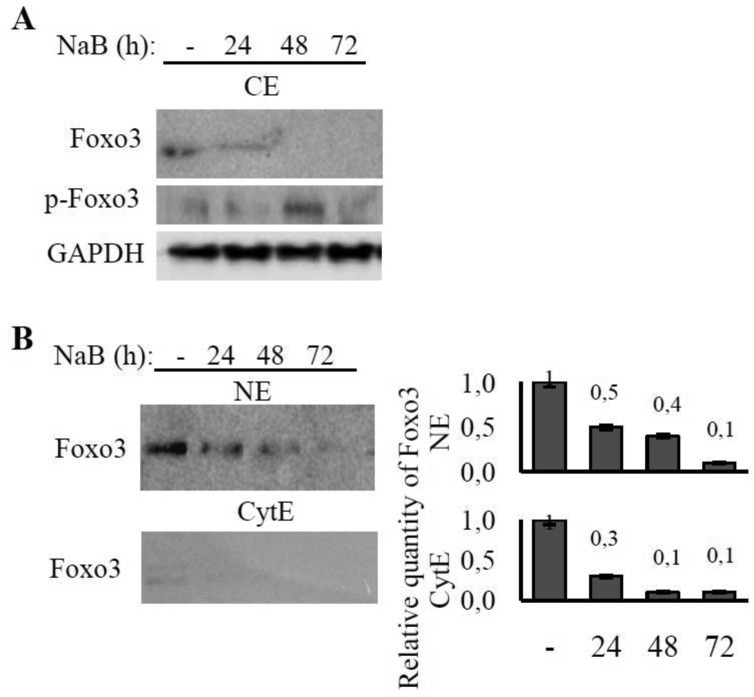
The effect of NaB treatment on FoxO3 expression and localization in E1A+Ras cells. The cells were treated as described in Figure 3. Immunoblotting of proteins performed on total (CE) extracts (A), nuclear (NE) and cytoplasmic (CyE) extracts (B) of E1A+Ras cells using antibodies against Foxo3 (A,B) and p-Foxo3 (Ser253) (A). Expression of GAPDH was used as loading control. The histograms under the immunoblot figures represent the calculation of WB bands intensity in relation to intensity in control point (-).

In order to evaluate FoxO modulation with NaB in E1A+Ras cells, the level of *foxo* transcription and total FoxO content in cells treated with NaB for 24–72 hours were measured. The study shows that prolonged NaB treatment causes only a slight decrease in *foxO1* mRNA level (Figure 3A). Therefore, NaB doesn't seem to have significant inhibitory effect on FoxO1 expression on the transcriptional level. At the same time, the FoxO1 and FoxO3 proteins level are declining significantly under prolonged NaB treatment (Figures 3B and 4A). It's interesting that NaB affect FoxO1 and FoxO3 proteins amount in different ways. FoxO1 accumulates dramatically after 24 hours of NaB treatment. But at 48 hours of treatment FoxO1 protein is undetectable. For its part, the level of FoxO3 is decreasing gradually.

The immunoblots show accumulation of FoxO1 after the first 24 hours with subsequent reduction in FoxO level. Possible explanations for this effect can be found in the discussion section.

The content of FoxO3 has declined within 24 hours of NaB treatment. This declining can be explained, apparently, by FoxO3 degradation in response to NaB-induced phosphorylation of FoxO3 by PKB/Akt kinase. The immunoblot of phosphorylated FoxO3 represents that phosphorylated form of FoxO3 shows up after 24 hours of NaB treatment (Figure 4A).

Other tumor cell lines with overexpression of Ras or both Ras and E1A have been used to demonstrate the specificity of NaB-dependent inhibition of FoxO. As well as in E1A+Ras cell line, in HEK-293 cells, expressing both mutant Ras and E1A, FoxO1 is degrading under NaB treatment but with less intensity (Figure S2). This can be possibly explained with higher E1A expression in HEK-293, since E1A is capable of preventing FoxO from degradation.

In contrast to E1A+Ras cells, cell lines which express the mutant Ras only (A-549, HCT-116) initially have lower FoxO1 protein level. In the absence of E1A constitutive Ras-induced Akt activation leads to intense FoxO degradation, which explains the difference in basal FoxO1 level. However, we observed straight accumulation of FoxO1 protein under NaB treatment in time dependent manner in HCT-116 and A-549 cancer cells with Ras mutation (Figure S2). The obtained data proves that E1A oncoprotein plays an important role in the NaB-dependent modulation of Foxo expression rather than the activated Ras.

### 3.3. NaB triggers nuclear shuttling of the FoxO proteins in E1A+Ras-transformed cells

According to the immunoblotting data performed on nuclear and cytoplasmic lysates, FoxO1 is evenly distributed between the nucleus and the cytoplasm of control untreated cells (Figure 3C). Prolonged NaB treatment leads to FoxO1 is detected mainly in nuclei and still stays stable at 48 hours. The cytoplasmic level of FoxO1 shows the following dynamic—the protein accumulates significantly within 24 hours and then degrades almost totally after 48 hours of treatment. In sum, the total FoxO1 level falls during the treatment with almost complete disappearance of cytoplasmic FoxO1, while nuclear FoxO1 is maintained at relatively stable level (Figure 3C).

The immunobloting data performed with nuclear, cytoplasmic and total cell extracts, reveal that a decrease of the total FoxO3a protein quantity under NaB treatment is accompanied by the redistribution of FoxO3a into the nucleus (Figure 4). The immunofluorescence data confirm the increase in the share of nuclear FoxO3a following NaB action (Figure S3). Immunofluorescent Foxo3 has been detected in nuclei both within 24 and 48 hours of NaB treatment.

**Figure 5.**
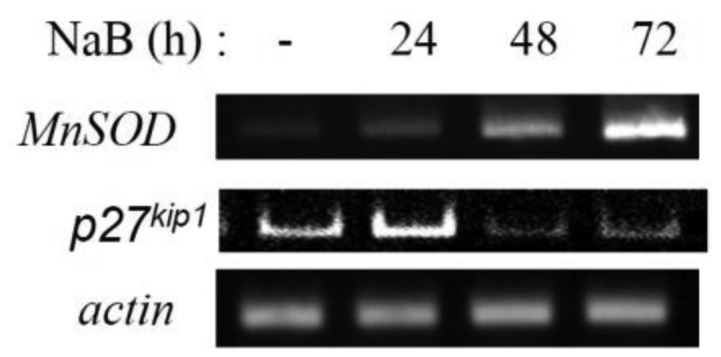
The effect of NaB on the transcription of the ROS scavengers in transformed cells E1A+Ras. RT-PCR analysis of MnSOD and p27^kip1^ transcription in cells untreated (-) or treated with NaB for 24–72 h. Actin gene transcription serves as a loading control.

The expression of FoxO-target genes coding for ROS scavengers, providing protection against oxidative stress, has been shown to increase under NaB treatment (Figure 5).

### 3.4. NaB stimulates additional accumulation of PKB/Akt kinase in E1A+Ras-transformed cells

**Figure 6.**
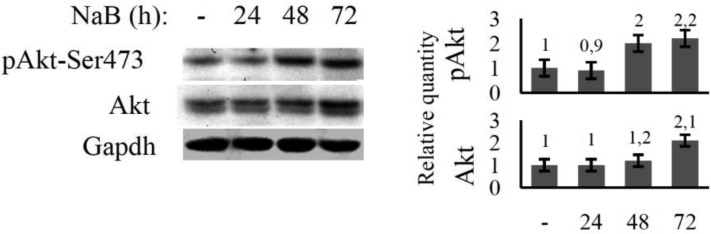
The effect of NaB on PKB/Akt kinase activity in transformed cells. Immunoblotting of proteins using antibodies against phosphorylated kinase Akt (Ser473) and total Akt in lysates of E1A+Ras transformed cells untreated (-) or treated with NaB for 24–72 h. Expression of GAPDH was used as loading control. The histogram under the immunoblot figure represents the calculation of WB bands intensity in relation to intensity in control point (-).

PKB/Akt kinase is a key mediator of Ras-induced ROS. There are several lines of evidence supporting that FoxO inhibition is important for Akt-induced ROS accumulation. As it was mentioned above, PKB/Akt kinase is constitutively active in E1A+Ras-transformed cells due to expression of cHa-ras oncogene [14]. Beyond that, lengthy NaB treatment has been shown to cause additional phosphorylation of PKB/Akt at Ser473 (Figure 6). The maximal phosphorylation level of PKB/Akt was reached after 48 h of NaB administration. The phosphorylation of Ser473 along with Thr308 provides PKB/Akt activation [18]. Thus, NaB seems to trigger activating phosphorylation of PKB/Akt. The dynamic of PKB/Akt phosphorylation correlates with phosphorylation of its target FoxO proteins and FoxOs degradation (Figures 3 and 4).

## 4. Discussion

The study has revealed that initially sodium butyrate has stimulating effect on FoxO1, but by prolonged exposure it causes degradation of FoxO through additional phosphorylation of PKB/Akt. This initial activation of FoxO1 can be presumably explained by a slight increase in ROS-level, triggering expression of the scavengers of ROS. The PCR results are also support this assumption. In some researches MnSOD was shown to be regulated directly through FoxO3a [19],[20], while in others FoxO1 is presented to be the key MnSOD regulator [21]. Some increase in the expression of MnSOD after 24 hours of NaB treatment is consistent with FoxO3 translocation to the nuclei. Summing up, activation of ROS scavengers transcription within first hours of NaB treatment, seem to be FoxO-dependent. However, further accumulation of ROS scavenger MnSOD can't be explained by activation of FoxO, since FoxO transcription factors undergo degradation during long-term NaB treatment, because of potent activation of PKB/Akt. Interestingly, one of the possible reasons of this dramatic accumulation of the ROS scavengers stems from PKB/Akt activation itself. As it was already mentioned, PI3K/Akt pathway results in NF-κB activation, which in turn has been shown to induce MnSOD expression [22]. Thus, antioxidant function in cells is not disrupted by FoxO degradation, since NF-κB is also capable of restraining ROS accumulation in the absence of FoxO, because many genes coding for antioxidant proteins, are NF-kB target genes [22].

Regulation of FoxO stability takes place through phosphorylation of FoxO by PKB/Akt kinase. PKB/Akt is a key mediator of Ras-induced ROS [23]. As we have shown NaB enhanced activating phosphorylation of PKB/Akt. But precise mechanism of NaB-dependent activation of PKB/Akt is still to be discovered. HDACIs alter genes expression, in part, through inhibition of histones deacetylation [24]. The point is, PKB/Akt can be activated either through elevated expression of kinase directly phosphorylating PKB/Akt, or through activation of any upstream regulator in this pathway. It's known that constant activity of PKB/Akt is one of the reasons of general FoxO inhibition, for instance in tumor development [2]. It therefore follows that the FoxO level in untreated E1A+Ras cells is initially lower than in original MEF line. NaB treatment result in supplemental stimulation of already activated PKB/Akt. PKB/Akt has been shown to activate oxidative metabolism by increasing oxygen consumption in mitochondria [25]. Accumulating under prolonged NaB treatment, Akt inactivates FoxO and facilitates ROS accumulation.

## 5. Conclusion

To summarize, our data show that NaB regulates FoxO transcription factors on the post-transcriptional level, in time-dependent manner, activating FoxOs and triggering their nuclear translocation after short-term treatment. But the general pattern of FoxO activation is being eroded by concomitant activation of PKB/Akt, which induces FoxO degradation at longer treatment. There are several lines of evidence supporting that FoxO inhibition is important for Akt-induced ROS accumulation and senescence [26]. These data are agreed with observation that only long-term treatment of E1A+Ras cells with HDACIs leads to accumulation of the features of NaB-induced senescence, including irreversibility of cell cycle arrest, hypertrophy, γH2AX foci accumulation and SA-βGal staining [27]. Previously we have shown that HDACIs induced irreversible cell cycle arrest and senescence in E1A+Ras cells. Specific increases of ROS level have been demonstrated as potentially critical for induction and maintenance of cell senescence process. So, we can conclude that one of the mechanism involved in HDACIs-induced cell senescence involves inhibition of FoxO transcription factors, leading to ROS excess generation.
