# Correction: Time-dependent modulation of FoxO activity by HDAC inhibitor in oncogene-transformed E1A+Ras cells

**DOI:** 10.3934/genet.2018.3.191

**Published:** 2018-09-13

**Authors:** Alisa Morshneva, Olga Gnedina, Svetlana Svetlikova, Valery Pospelov, Maria Igotti

**Affiliations:** Institute of Cytology, Russian Academy of Sciences, St. Petersburg, Russia

We would like to submit the following correction to our recently published paper [1] due to the incorrect information in the Acknowledgments section by the authors' mistake. The correct Acknowledgments are provided below:

**Acknowledgements**

This work was supported by Russian Science Foundation (RSF) under Grant No. 14-50-00068.

The changes have no material impact on the conclusion of this article. The original manuscript will be updated. We apologize for any inconvenience caused to our readers by this change.
