# Supplementary material for: Time-dependent modulation of FoxO activity by HDAC inhibitor in oncogene-transformed E1A+Ras cells
Source: AIMS Genet. 2018 Feb 8;5(1):41–52. doi: 10.3934/genet.2018.1.41 (PMC6690250; doi:10.3934/genet.2018.1.41)
Supplement: Supplementary file 1 [file genetics-05-01-041-s01.pdf]

AIMS Genetics

AIMS Genetics, 5(1): 41–52.
DOI: 10.3934/genet.2018.1.41
Received: 01 November 2017
Accepted: 01 February 2018
Published: 08 February 2018

http://www.aimspress.com/journal/Genetics

Research article

Time-dependent modulation of FoxO activity by HDAC inhibitor in oncogene-transformed E1A+Ras cells

Alisa Morshneva, Olga Gnedina, Svetlana Svetlikova, Valery Pospelov and Maria Igotti*

Institute of Cytology, Russian Academy of Sciences, St. Petersburg, Russia

* **Correspondence:** Email: marie.igotti@gmail.com; Tel: +78122973740; Fax: +78122970341.

Supplementary

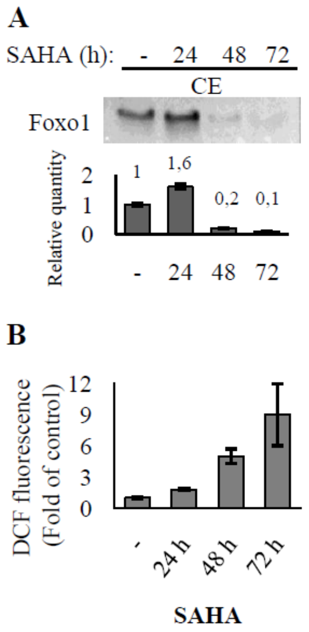

Figure S1. The effect of HDAC inhibitor SAHA on FoxO1 expression in E1A+Ras cells. The cells were treated with 2.5 μM SAHA for 24–72 h whilst one well was left

untreated (-) and used as a control. (A) Immunoblotting of proteins performed on total extracts using antibodies against Foxo1. The histograms under the immunoblot figures represent the calculation of WB bands intensity in relation to intensity in control point (-). (B) ROS levels as measured by FACS analysis after staining with DCFDA. The augmentation of median X meaning due to increased fluorescence indicates an increase in the intracellular levels of ROS. The results are presented as the ratio of mean DCF fluorescence at each experimental point to the mean fluorescence in control untreated cells. DCF fluorescence in untreated cells is taken as unit.

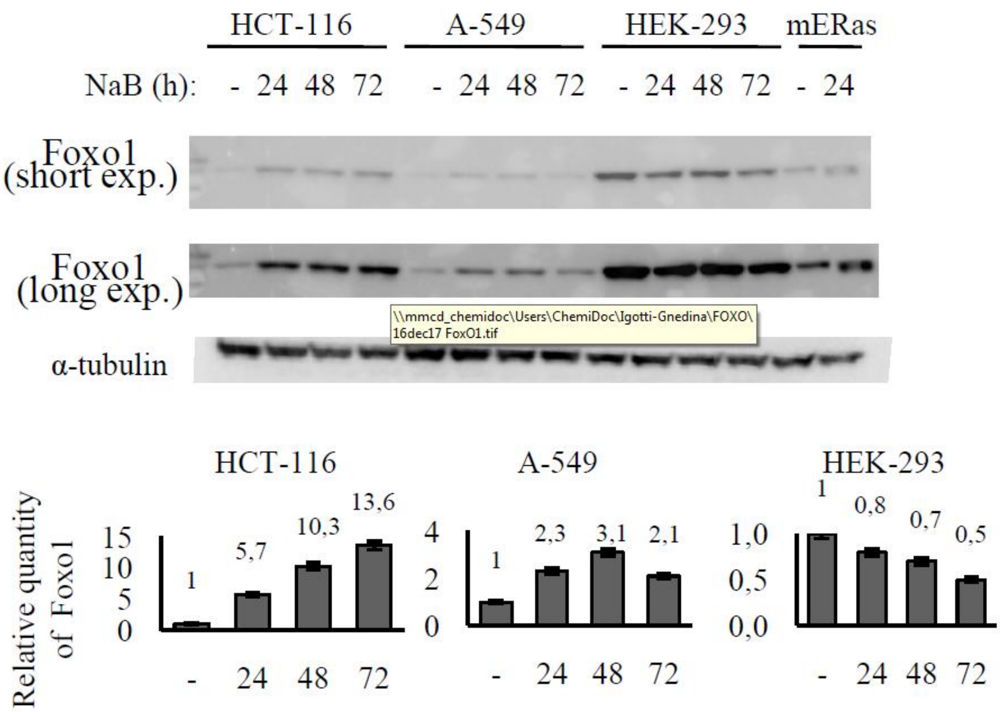

Figure S2. The effect of NaB treatment on FoxO expression in human tumor cells. The cells HCT-116, A-549, HEK-293 amd E1A+Ras were treated with 4 mM NaB for 24–72 h whilst one well was left untreated (-) and used as a control. Immunoblotting of proteins performed on total extracts using antibodies against Foxo1. Expression of α-tubulin was used as loading control.

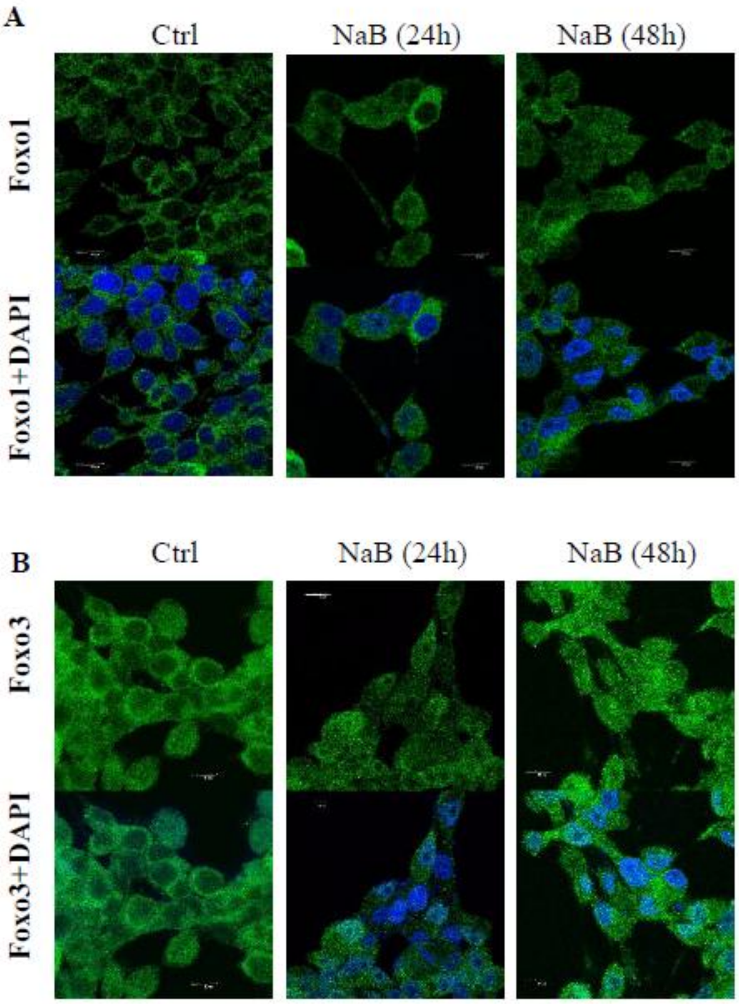

Figure S3. The influence of NaB treatment on the cell localization of the FoxO family proteins in cells E1A+Ras. The cells were treated as described in Figure 3. Immunofluorescence analysis of Foxo1 (A) and Foxo3 (B) proteins localization.

AIMS Press

© 2018 the Author(s), licensee AIMS Press. This is an open access article distributed under the terms of the Creative Commons Attribution License (http://creativecommons.org/licenses/by/4.0)
